# A univocal definition of the neuronal soma morphology using Gaussian mixture models

**DOI:** 10.3389/fnana.2015.00137

**Published:** 2015-11-03

**Authors:** Sergio Luengo-Sanchez, Concha Bielza, Ruth Benavides-Piccione, Isabel Fernaud-Espinosa, Javier DeFelipe, Pedro Larrañaga

**Affiliations:** ^1^Computational Intelligence Group, Departamento de Inteligencia Artificial, Escuela Técnica Superior de Ingenieros Informáticos, Universidad Politécnica de Madrid, Campus MontegancedoMadrid, Spain; ^2^Laboratorio Cajal de Circuitos Corticales, Centro de Tecnología Biomédica, Universidad Politécnica de Madrid, Campus MontegancedoMadrid, Spain; ^3^Consejo Superior de Investigaciones Científicas, Instituto CajalMadrid, Spain; ^4^Centro de Investigación Biomédica en Red Sobre Enfermedades Neurodegenerativas, Instituto de Salud Carlos IIIMadrid, Spain

**Keywords:** three-dimensional soma reconstruction, repair and segmentation, morphology validation, mesh comparison, Gaussian mixture model

## Abstract

The definition of the soma is fuzzy, as there is no clear line demarcating the soma of the labeled neurons and the origin of the dendrites and axon. Thus, the morphometric analysis of the neuronal soma is highly subjective. In this paper, we provide a mathematical definition and an automatic segmentation method to delimit the neuronal soma. We applied this method to the characterization of pyramidal cells, which are the most abundant neurons in the cerebral cortex. Since there are no benchmarks with which to compare the proposed procedure, we validated the goodness of this automatic segmentation method against manual segmentation by neuroanatomists to set up a framework for comparison. We concluded that there were no significant differences between automatically and manually segmented somata, i.e., the proposed procedure segments the neurons similarly to how a neuroanatomist does. It also provides univocal, justifiable and objective cutoffs. Thus, this study is a means of characterizing pyramidal neurons in order to objectively compare the morphometry of the somata of these neurons in different cortical areas and species.

## 1. Introduction

Vertebrate neurons generally show a morphological and functional polarization so that neurons can be divided into separate regions: a receptive and conductive apparatus (formed by the dendrites and cell body or soma), an emission apparatus (the axon), and a distribution apparatus (terminal axonal arborization). Neurons may receive particular inputs from their dendrites (proximal vs. distal), dendritic structures (dendritic spines vs. dendritic shafts), and cell body. Furthermore, the axon initial segment of some neurons receives inputs either in its proximal portion or along its length (e.g., Peters et al., 1991). The cell body of the neuron contains a large, spheroidal nucleus (with one or more nucleoli) containing a nuclear membrane and a highly differentiated cytoplasm (perikaryon). The cell body, dendrites and axon have distinct physiological and molecular characteristics (e.g., Szu-Yu Ho and Rasband, 2011), and these compartments can in general terms be identified morphologically or neurochemically; for example, IκBα immunostaining recognizes an unidentified protein associated with the microtubule-based cytoskeleton at the axon initial segment (Buffington et al., 2012) and can be used to demarcate the axon initial segment (e.g., Schultz et al., 2006; Sánchez-Ponce et al., 2012).

To our knowledge, there is no line demarcating the soma of the labeled neurons and the origin of the dendrites and axon. Thus, the morphometric analysis of the neuronal soma is highly subjective. Differentiating between these compartments and delimiting the neuron cell body is usually a job for neuroanatomists, which they do according to their own arbitrary criteria, as it is not absolutely clear what constitutes the cell body of the labeled neurons. Since morphological measures rely directly on the delimitation of the cell body, different neuroanatomists segmenting the same neuron might get different somatic sizes and shapes. Thus, the results of different researchers are inaccurate and hard to compare. Furthermore, high-throughput imaging methods have expanded quickly over the last few years, and the manual tracing of individual cells is a time-consuming task. Thus, it is necessary to develop automatic techniques to acquire morphometric data on labeled neurons. Ideally, the morphometric analysis of the cell bodies should be performed automatically on complete 3D reconstructions of cells using specialized algorithms. 3D reconstructions from image stacks can be quite easily performed using a variety of techniques, including confocal microscopy to reconstruct, for example, certain types of neurons from transgenic animals in which neurons are labeled with green fluorescent protein, or from brain tissue where neurons have been labeled after fluorescent dyes. Here we selected cortical pyramidal cells, which are the most abundant and characteristic neuronal cell type in the cerebral cortex (DeFelipe and Fariñas, 1992). We used cells that were intracellularly injected with Lucifer Yellow as part of an unrelated research (Benavides-Piccione et al., 2013). In the present study we generated the 3D reconstruction of the soma of the neuron and proximal dendrites. The 3D generated somata sometimes showed distortions due to the hole produced by the micropipette used to inject the dye. Thus, the labeled cell bodies are not suitable for an automated morphological analysis because the measurements on a damaged surface are incorrect.

**Figure 1:**
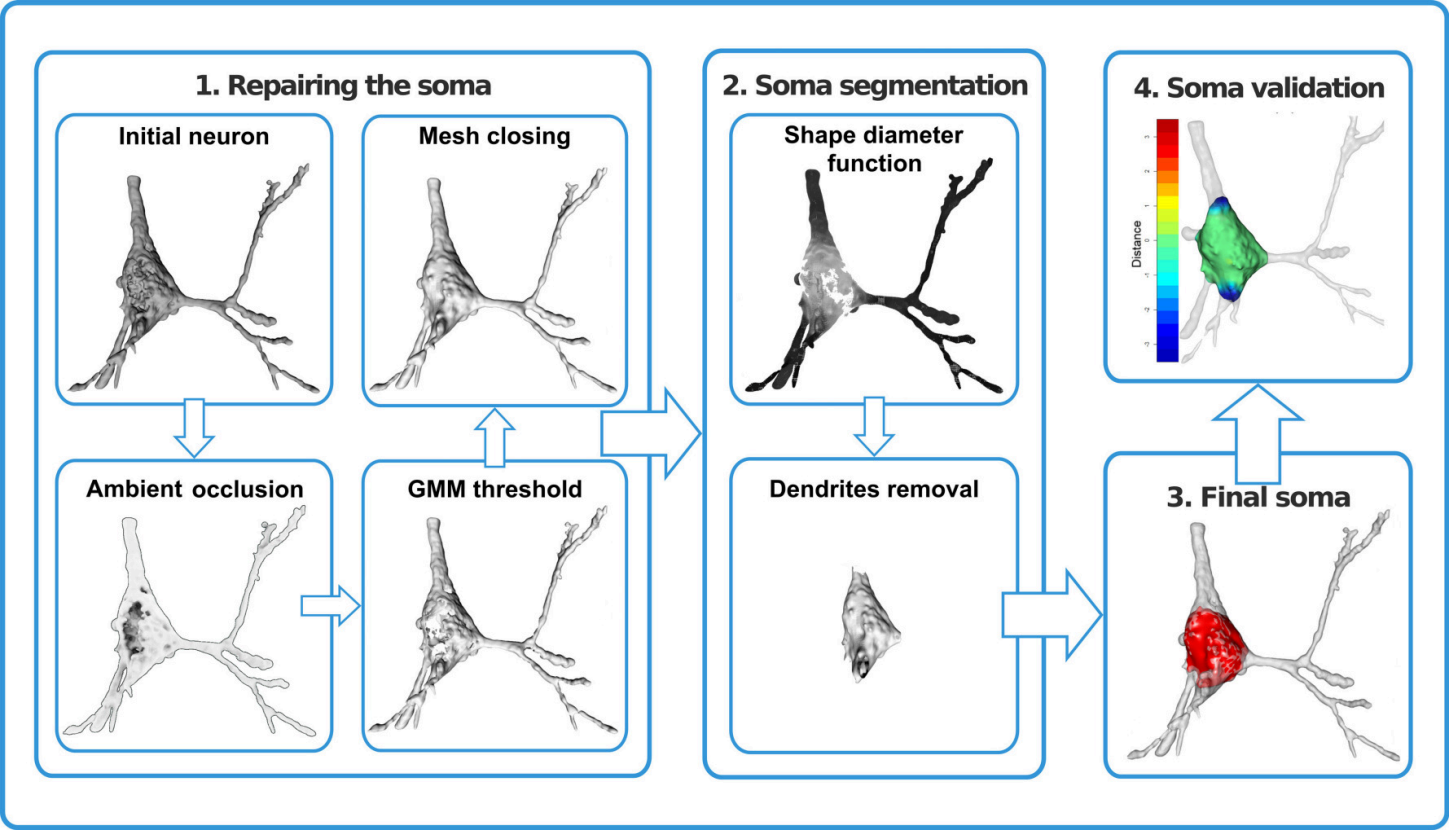
**Repair and segmentation process workflow**. The process was divided into four modules. First, the neuron was repaired by removing holes and cavities and closing the mesh. Second, dendrites were identified and removed. As a result, the neuronal cell body was defined (third module) and was validated (fourth module) by means of distance computations and volume comparisons.

In this article, we propose a procedure for repairing the three-dimensional virtualized cell bodies. We also introduce a mathematical method combining probabilistic clustering and 3D mesh processing algorithms to provide a univocal, justifiable, and objective characterization of how a soma can be defined. This method should help researchers to establish and maintain effective communication and data sharing (Figure 1).

## 2. Methods and results

Neurons were intracellularly injected with Lucifer Yellow (LY) in layer III of the human cingulate (25 somas), temporal (16 somas), and frontal (18 somas) cortex from a 40-year-old human male obtained at autopsy (2–3 h post-mortem). Further information regarding tissue preparation, injection methodology, and immunohistochemistry processing is given in Benavides-Piccione et al. (2013). Stacks of images (63x) including pyramidal cell somata and their proximal dendrites were acquired using a Leica TCS 4D confocal scanning laser attached to a Leitz DMIRB fluorescence microscope. Somata were reconstructed in 3D using Imaris software 7.6.4 yielding, by thresholding, a solid surface that matched the contour of the neuron. The generated surface, called triangular mesh, was composed of two basic elements, vertices which defined three-dimensional Cartesian points and faces that denoted the edges between vertices. Each face was a set of three edges connecting vertices forming a triangle of the triangular mesh.

### 2.1. Repairing the soma

**Figure 2:**
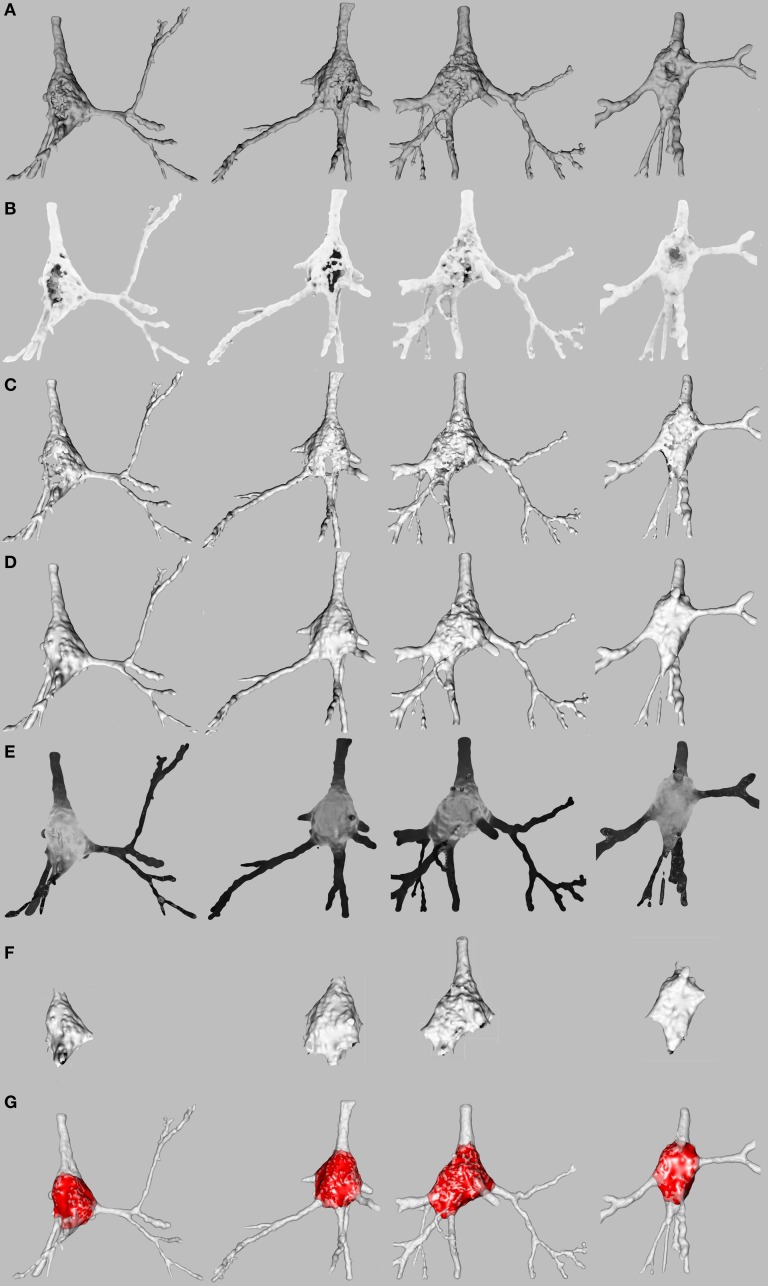
**Repair and segmentation process**. **(A)** Initial state of four representative pyramidal cells. **(B)** Neuron exposure to ambient lightning. **(C)** Neuron after vertices forming holes and cavities or positioned inside the mesh have been discarded. **(D)** Neuron after mesh closing. **(E)** Vertices of the mesh colored according to shape diameter function to segment soma and dendrites. **(F)** Neuron after the basal dendrites have been removed. **(G)** Final result.

Soma surfaces frequently showed faults like holes or cavities produced by the intracellular injection procedure (Figure 2A). MeshLab software (CNR, 2008) was used for the purposes of both repair and segmentation by means of automatic scripts of MeshLab.

The faults on the surface were regarded as noise which should be removed. An approximation of the original shape of the soma was then computed to achieve a single closed mesh. We called this process “repairing the soma” (Figures 2B–D). The first step in the repair process consisted of distinguishing between the vertices on the surface and the vertices forming holes, cavities or placed inside the neuron.

Assuming that a neuron could be isolated in a fictitious lighting space, the vertices of the neuron on the mesh surface would be exposed to light, whereas the vertices that formed a hole or were placed inside the mesh would be darkened. Thus, light exposure information has the potential to distinguish between the vertices forming the original surface of the neuron and the vertices introduced by the injection.

**Figure 3:**
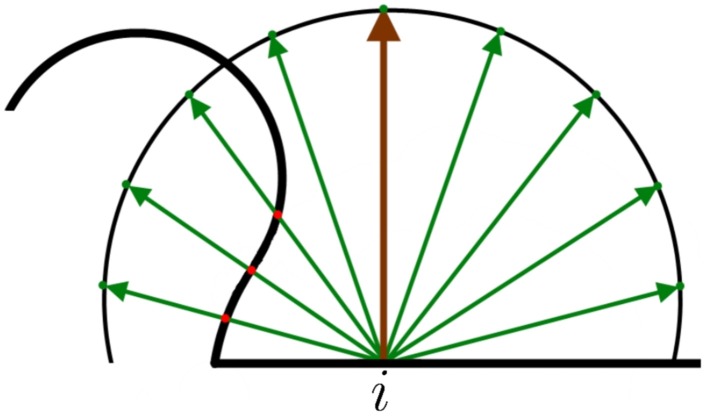
**Example of 2D ambient occlusion**. The surface of the mesh is the thick black line. The brown line denotes the normal vector of the evaluated vertex *i*. A hemisphere is placed around the normal vector. The green lines represent the sampled *N* points of the hemisphere. The red dots are the intersection between the rays and the mesh surface. In this case, occlusion factor $=\frac{3}{8}$.

This motivated the application of ambient occlusion (Zhukov et al., 1998) of MeshLab, which is a technique that provides a way to estimate the amount of light projected onto a vertex of a mesh through ray tracing. The ambient occlusion factor *A* is a measurement of the light rays blocked by the objects around the evaluated vertex. For each vertex, a hemisphere with an infinite radius oriented according to its own normal vector was generated (Figure 3). Then, *N* points of the hemisphere were sampled uniformly. Next, rays were traced from the evaluated vertex to each sampled point. Counting the number of rays that intersected the mesh surface (*N*_*i*_), obviously disregarding the starting point, and comparing it with the total number of traced rays (*N*), the ambient occlusion for a vertex *i* was computed as $A_{i}=\frac{N_{i}}{N}$ (see Figure 3).

The result of the scalar value *A*_*i*_ was in the range [0, 1], where 0 denoted that no ray intersected the surface of the mesh and 1 meant that all the traced rays intersected the mesh and consequently that the vertex was inside the mesh. The points whose ambient occlusion factor *A*_*i*_ was close to 0 were exposed to light and colored white and the points close to 1 were colored black (see Figure 2B).

Because some vertices were artifacts introduced by the filling process they had to be discarded. A simple approach could be to impose an arbitrary threshold such that vertices whose ambient occlusion factor was greater than the threshold would be discarded. However, the threshold should preferably be estimated automatically.

At this point, we considered that a clustering algorithm, whose goal is to group instances of similar data in the same group, fitted the problem specifications exactly. Probabilistic clustering based on a Gaussian mixture (McLachlan and Basford, 1988) was applied to cluster vertices into two groups: (i) the vertices on the surface of the neuron; (ii) the vertices forming holes and cavities or inside the neuron. Probabilistic clustering returned the probability of each vertex being a member of either cluster. The decision boundary between both clusters, i.e., the ambient occlusion factor for which both groups were equiprobable, was the threshold. Vertices *i* whose *A*_*i*_ factor was greater than the threshold were removed, as were their associated faces. As a consequence, the mesh was opened as shown in Figure 2C. An approximation of the original surface of the soma was computed to achieve a single closed mesh (Figure 2D) as explained below.

**Figure 4:**
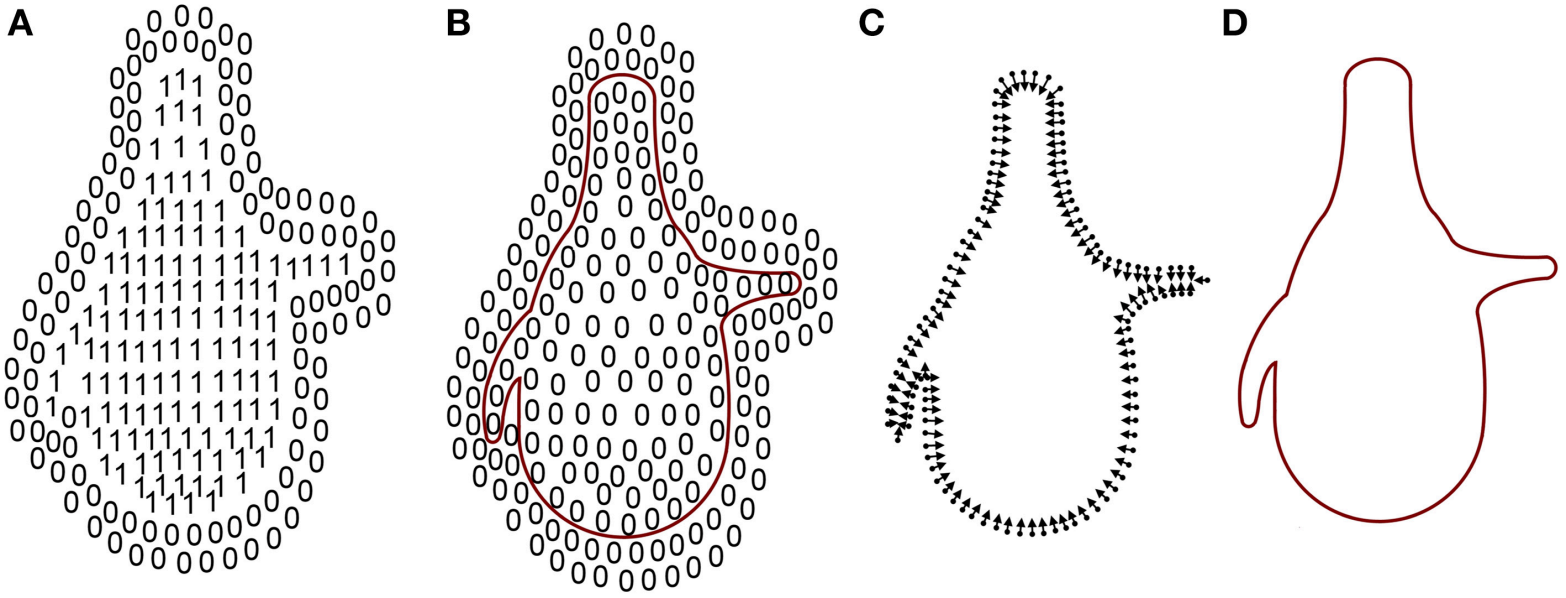
**Mesh reconstruction**. **(A)** The indicator function. **(B)** Inward-facing normals and their vertices. **(C)** Gradient of the indicator function. Since the indicator function is constant outside (0s) and inside (1s) the mesh, the gradient of the space there is 0. Only points on the frontier are not 0. **(D)** Surface of the mesh. Adapted from Kazhdan et al. (2006).

A simple way to define the closed surface of an object is by means of an indicator function that denotes the space inside and outside the object as 1 and 0, respectively (Figure 4A). Thus, as a result of computing the gradient of this function, space would be zero almost everywhere except near the surface of the object (Figure 4B).

However, the indicator function was unknown and only the vertices and the inward-facing normals of the mesh were provided by Imaris and MeshLab software (Figure 4C). A relationship between an integral of the surface normal field and the gradient of the indicator function was derived in Kazhdan et al. (2006). Unfortunately, this relationship could not be exploited since the surface geometry was unknown so the integral of the surface normal field could not be computed. In Kazhdan et al. (2006) this problem was solved by means of an approximation of the integral with a discrete summation over the vertices and the inward-facing normals of the mesh (Figure 4D). Applying this method, known as Poisson surface reconstruction, the holes in the surface introduced in the previous step disappeared and the surface of the soma was also slightly smoothed.

### 2.2. Automatic soma segmentation

Finally, segmentation can be understood as a clustering problem where each vertex belongs to one cluster, either soma or dendrite.

**Figure 5:**
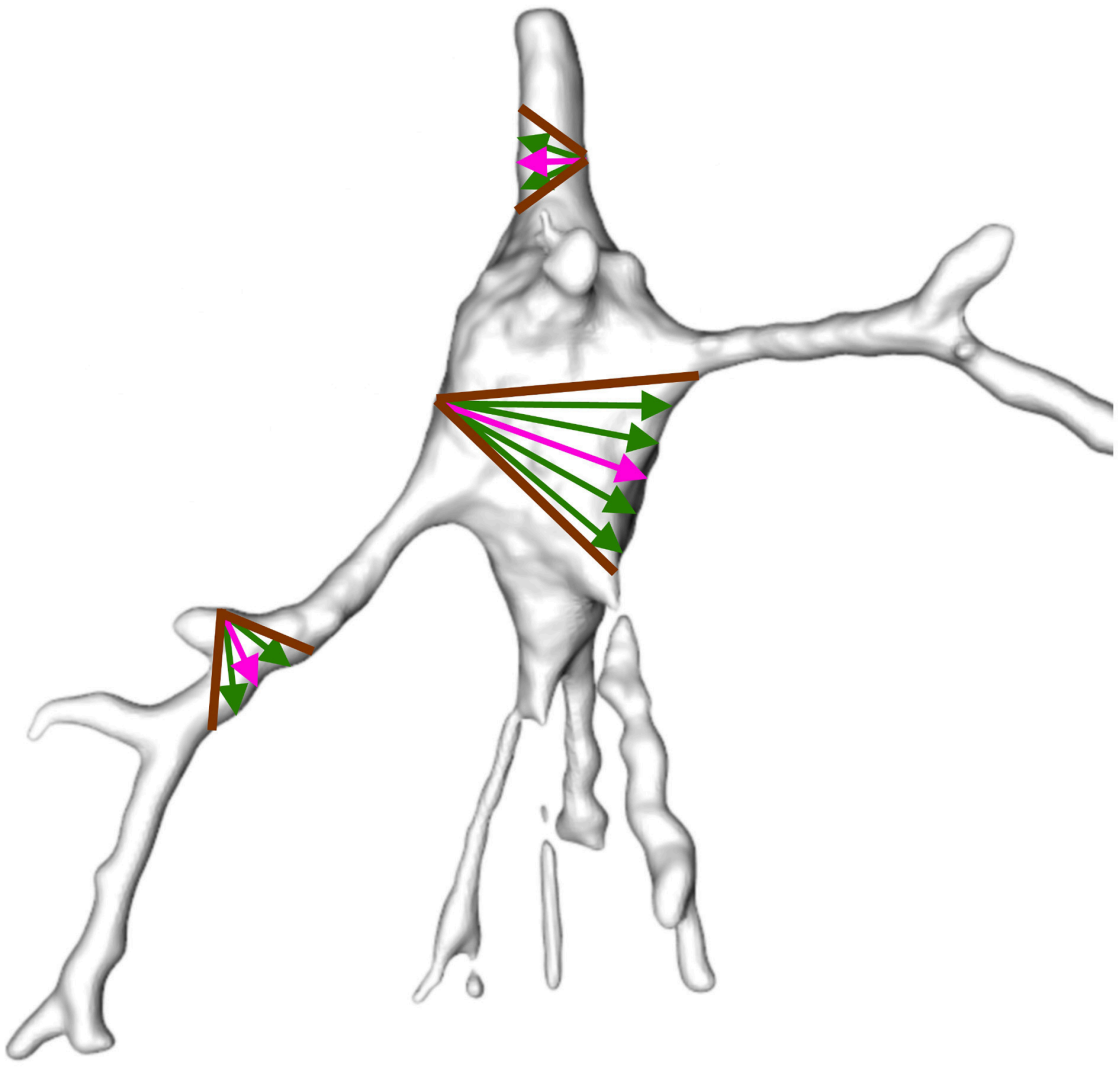
**Example of shape diameter function**. A cone (brown) is centered on the inward-normal of each vertex (pink arrow). Several rays (green) are sampled inside the cone such that the sum of the length of the rays from the vertex to their intersection with the mesh surface on the opposite side of the mesh approximates vicinity volume. The rays sampled inside the soma are longer than the rays sampled inside the dendrites and the volume of the vertices in the vicinity of the soma is therefore greater.

Shapira et al. (2008) presented a scalar function, called shape diameter function (SDF), based on exploiting differences between the volume in the neighborhood of the vertices of the mesh. This property is suitable for segmentation since dendrites are thinner than the soma and the volume in the vicinity of the vertices of the soma is therefore greater. An illustration of SDF computation for some mesh vertices is shown in Figure 5.

The gray-shaded neurons illustrated in Figure 2E were obtained from the SDF outcome. The vertices of the mesh were colored according to the value of the scalar function SDF such that the darker the vertex, the smaller the vicinity volume. Consequently, the vertices of the somata were gray, and the vertices of the dendrites were black.

**Figure 6:**
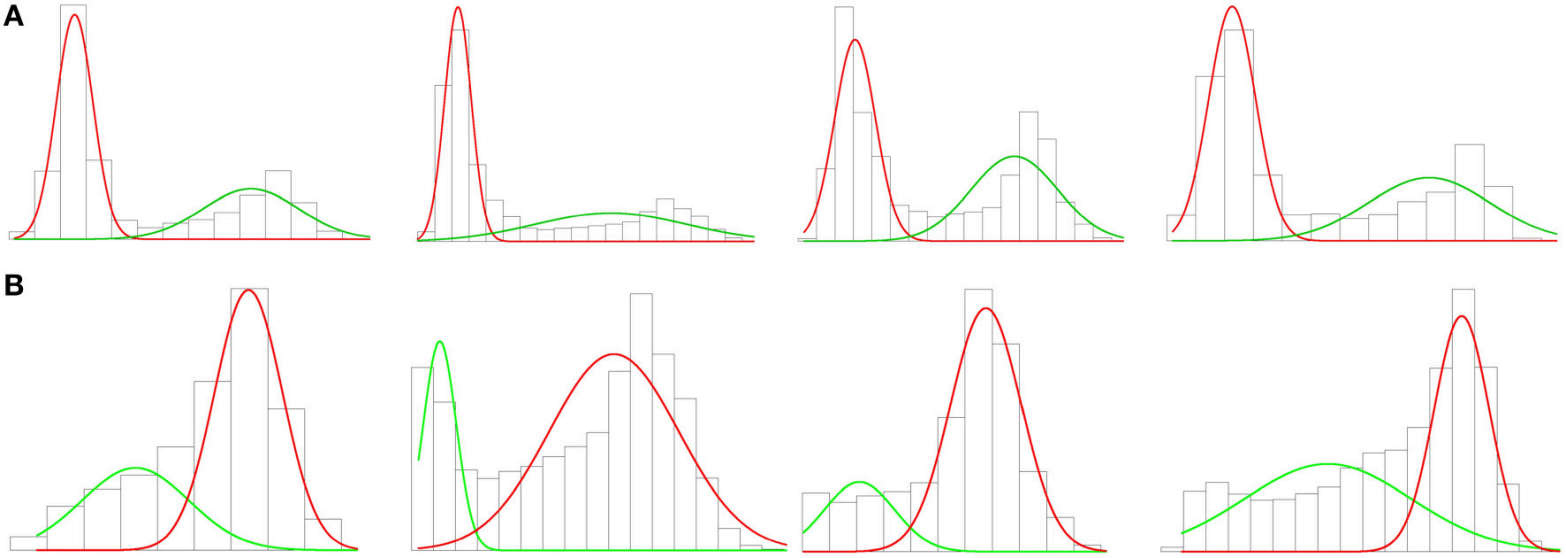
**(A)** Histogram and first clustering. The charts represent the volume distribution of the soma and the apical dendrite (red) and the basal dendrites (green) of the neurons shown in Figure 2. There are clearly two Gaussians. **(B)** Histogram and second clustering. The charts show the volume distributions in the vicinity of the soma (red) and the apical dendrite (green) of the outcome of the first clustering. The graphs columnwise represent the same cell along the process. There are also two Gaussians in these new graphs, demonstrating that the apical dendrite was hidden. This clustering removes the vertices of the apical dendrite in some cases, as in the second chart, and improves the accuracy of the cutoffs in other cases, as in the fourth chart.

As with ambient occlusion, some vertices were discarded. In this case, the vertices of the soma were kept whilst vertices of the dendrites were removed. Thus, a threshold based on the SDF outcome was imposed. Again probabilistic clustering based on a Gaussian mixture was applied to build a mathematical model for vertex clustering. The one-dimensional distribution of the SDF outcome appeared to fit a two-component Gaussian mixture (see Figure 6A), the soma and the dendrites. However, since the apical dendrite is typically thicker than the basal dendrites, sometimes the clustering algorithm regarded the apical dendrite as part of the soma. So, we tried clustering into three groups: apical dendrite, basal dendrites, and soma. We did not succeed, since in those cases where the apical and basal dendrites were quite similar, both dendrites were grouped in the same (first) cluster. The vertices of the soma were assigned 50–50 into two (second and third) clusters, cutting the soma by half. The observed problems in identifying the neuron regions were due to the fact that there were far fewer vertices representing the apical dendrite than there were for the soma or the basal dendrites. Because the volume of the apical dendrite was between that of the soma and basal dendrites, it did not show up in the histograms, and only two Gaussians were noticeable in Figure 6A, one for the soma and the other for the basal dendrites.

In order to overcome this problem, we defined a two-step process. In the first step, we separated out basal dendrites from apical dendrite and somata by means of two Gaussian clustering according to the SDF distribution (Figure 6A). Thus, the vertices and the faces of the mesh which belonged to the basal dendrites were automatically identified and discarded (Figure 2F). In the second step, two Gaussian clustering was applied to distinguish between the soma and the apical dendrite (Figure 6B). The vertices and faces of the apical dendrite were identified and discarded by segmenting the soma. The apical dendrite was sometimes removed in the first step; the second clustering step improved cutoff accuracy in such cases.

**Figure 7:**
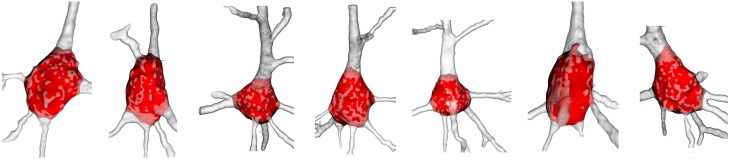
**Examples of final soma result**. The reconstructed neuron is colored white with low opacity and its automatically extracted soma is denoted in red. The surface of the extracted soma placed inside the dendrite is shaded light red.

The resulting soma was an open mesh and was then closed using the Kazhdan et al. (2006) method (Figure 2G). Other example of resulting somata, where the repaired and extracted soma is displayed and placed over the original neurons, are shown in Figure 7.

### 2.3. Mesh comparison

The distance between the surfaces of two triangular meshes quantifies the distortion added by a mesh processing technique. In our case, the distance was computed to validate the goodness of the proposed method.

The distance between two meshes is defined as the minimum distance from each point on the surface *S*_1_ of a mesh to the surface *S*_2_ of a second mesh. As the boundaries of a mesh are defined by its vertices, we studied the distance from vertices only. The distance ϵ between a vertex *p* ∈ *S*_1_ and the surface *S*_2_ was computed according to Cignoni et al. (1998) as

$$\begin{array}{l} \epsilon \left(p,S_{2}\right)=\underset{p^{\prime }\in S_{2}}{\min}d\left(p,p^{\prime }\right), \end{array}$$

where *d* is the Euclidean distance between *p* and *p*′ in ℝ^3^. Then the root mean square error (RMSE) was computed as follows:

$$\begin{array}{l} RMSE(S_{1},S_{2})=\sqrt{\frac{\sum_{p\in S_{1}}\epsilon {\left(p,S_{2}\right)}^{2}}{\left|S_{1}\right|}}, \end{array}$$

where |*S*_1_| is the number of vertices of the surface of the mesh. RMSE is an asymmetric measure as *RMSE*(*S*_1_, *S*_2_) ≠ *RMSE*(*S*_2_, *S*_1_). A symmetric form of the RMSE was obtained as

$$\begin{array}{l} RMSE_{S}\left(S_{1},S_{2}\right)=\max\left\{RMSE\left(S_{1},S_{2}\right),RMSE\left(S_{2},S_{1}\right)\right\}. \end{array}$$

Thus, *RMSE*_*S*_(*S*_1_, *S*_2_) = 0 ⇔ *S*_1_ = *S*_2_.

Also, it is useful to compare different mesh processing techniques. For two techniques, one approach was based on processing *M* meshes with each processing method. Then the volume of each processed mesh was calculated according to Zhang and Chen (2001). Mesh processing techniques were compared by the mean absolute quotient between volumes (*MAQ*). Its outcome was an estimation of the proportional difference in volume when a method is applied in place of the other:

$$MAQ_{1,2}=\frac{\sum_{i=1}^{M}\left|\frac{T_{1_{i}}}{T_{2_{i}}}-1\right|}{M},$$

where *T*_1_*i*__ is the volume of mesh *i* produced by the first technique, *T*_2_*i*__ is the volume of mesh *i* produced by the other technique and *M* is the total number of meshes processed by both methods. *MAQ* is also an asymmetric measure as *MAQ*_1, 2_ ≠ *MAQ*_2, 1_. A symmetric form of the *MAQ* was obtained as

$$\begin{array}{l} MAQ_{S}=\max\left\{MAQ_{1,2},MAQ_{2,1}\right\}. \end{array}$$

Thus, *MAQ*_*S*_ = 0 ⇔ *MAQ*_1, 2_ = *MAQ*_2, 1_.

### 2.4. Validation of automatic segmentation

**Figure 8:**
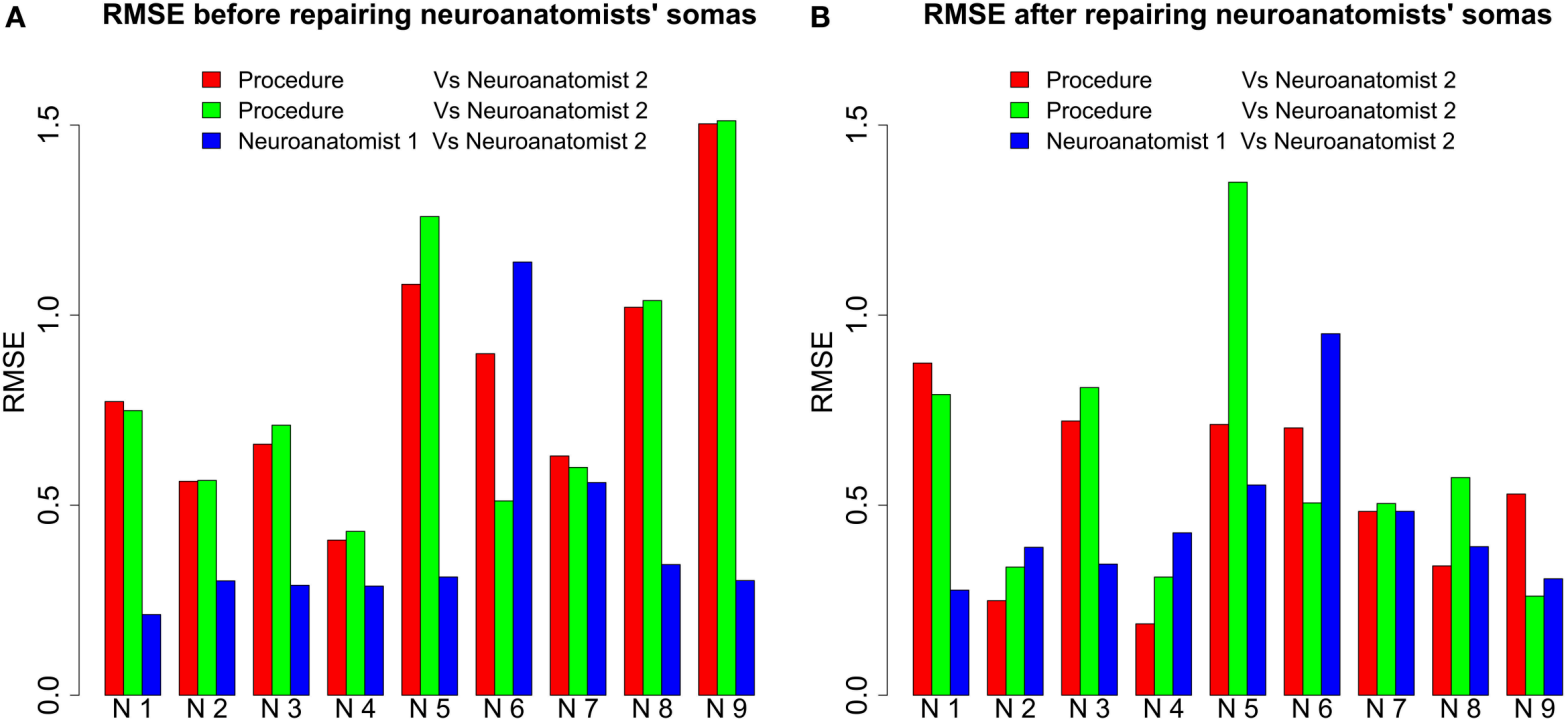
**RMSE before and after repairing the neuroanatomists' somata**. **(A)** For all neurons (each one denoted by N followed by a number) except Neuron 6, RMSE was less between neuroanatomists than between the somata output by our procedure and by either of the neuroanatomists. For several neurons, the difference was actually more than double. **(B)** The differences between automatically and manually segmented somata were not so remarkable after the repair of the neuroanatomists' somata. Note that for some neurons RMSE was less between our procedure and the first neuroanatomist than between both neuroanatomists, i.e., the proposed procedure can produce similar cutoffs to a neuroanatomist.

In order to validate the goodness of the automatic segmentation method, two neuroanatomists manually segmented nine three-dimensional neurons to set up a framework for comparison. Neuroanatomists used Imaris software. This software provides a three-dimensional representation of the neuron that can be rotated. It also allows to segment the soma cutting dendrites with geometrical planes. For all nine neurons, we compared the RMSE for the somata segmented manually by the neuroanatomists and the somata output by the automatic method whose surface had been repaired. The differences between both neuroanatomists' cutoffs, i.e., the inter-neuroanatomist variability, were also quantified (see Figure 8A).

The Wilcoxon signed-rank test was applied to corroborate the discrepancies in the plots observed in Figure 8A. It was assumed as a null hypothesis *H*_0_ that the RMSE between automatically and manually segmented somata was not significantly different from the inter-neuroanatomist RMSE. As a result, taking as reference the standard significant level of 0.05, *H*_0_ was rejected for the first neuroanatomist (*p* ≈ 0.02). Hence, there were found to be significant differences between the morphology of the first neuroanatomist's somata and the morphology of the somata yielded by the proposed procedure. Nevertheless, *H*_0_ could not be rejected for the second neuroanatomist (*p* ≈ 0.055).

**Figure 9:**
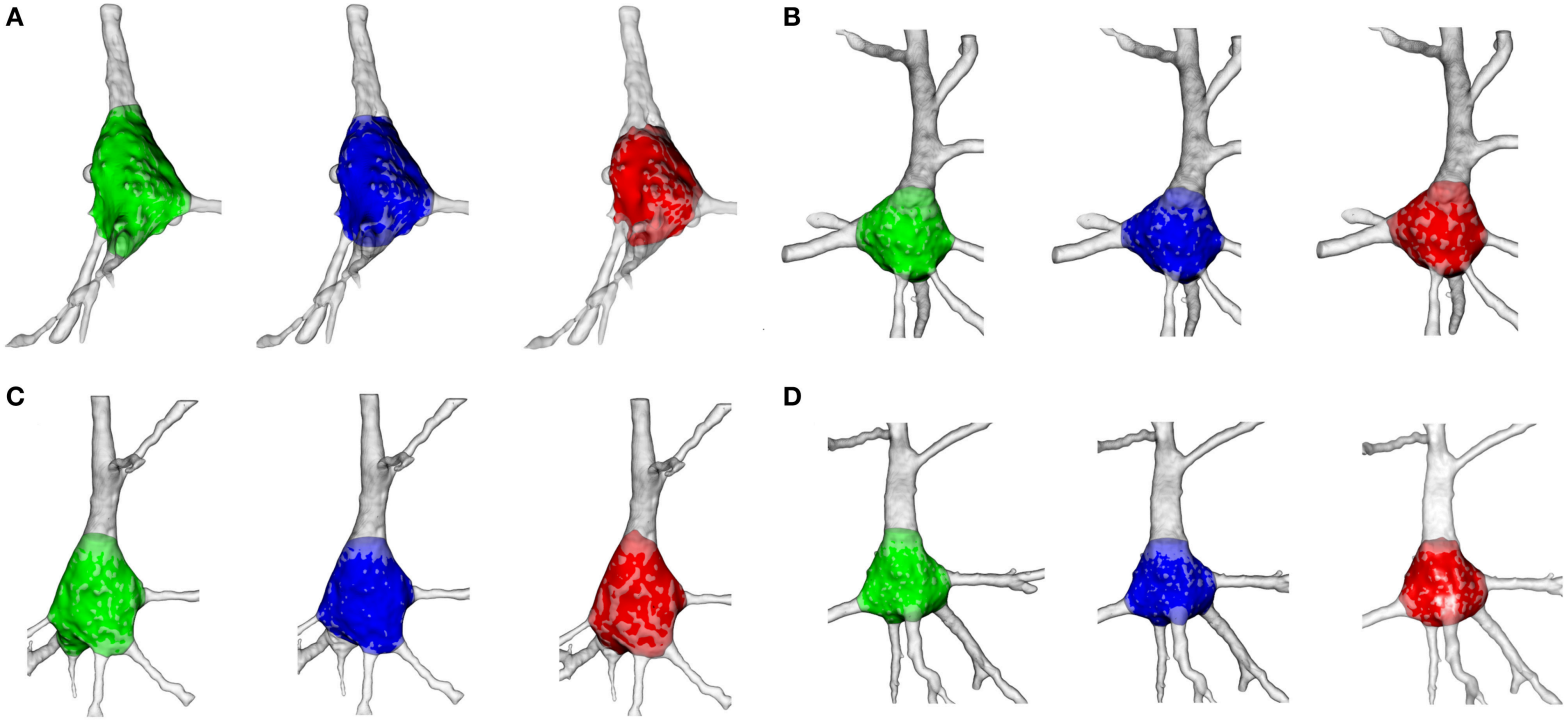
**Illustration of the goodness of the soma segmentation method on four cells**. The somata segmented manually by the first neuroanatomist are shaded green, the somata segmented by the second neuroanatomist are shaded blue and the somata segmented according to the proposed procedure are shaded red. **(A)** Segmentation of neuron 5. **(B)** Segmentation of neuron 9. **(C)** Segmentation of neuron 7. **(D)** Segmentation of neuron 6.

In the light of the findings of the Wilcoxon test, the neuroanatomists' somata were repaired to test whether the discrepancies with the automatically extracted somata were due to the method of repair or the segmentation process. Then three-dimensional representations of the somata were rendered (Figure 9). The resulting three segmentations for each neuron unveiled similar geometries, except for some fine distinctions in the cutoffs surfaced around the boundaries between the soma and apical dendrite. Hence, the significant differences previously observed between somata could be due to the repair process.

To find out this, the manually segmented somas were repaired by the automatic repair process and RMSE was recomputed. Figure 8B shows RMSE between the automatically and manually extracted somata. In this case, *H*_0_ was not rejected for either the first (*p* ≈ 0.73) or the second neuroanatomist (*p* ≈ 0.43). Hence, it was the repair process that caused the significant differences between the automatically and manually extracted somata.

We then calculated the *MAQ* between the volumes of the automatically and manually segmented somata. Thus, we found that there is a 4.33% and a 5.06% of difference in the volume of the somata segmented by the proposed process and the manually segmented somata by the first [*MAQ*_*S*_(*Proc, Exp*_1_)] and second neuroanatomist [*MAQ*_*S*_(*Proc, Exp*_2_)], respectively. Consequently, the difference in the volume of the somata between the procedure and the neuroanatomists was on average around a 4.7%. As regards somata segmented by neuroanatomists, the inter-neuroanatomist difference in volume [*MAQ*_*S*_(*Exp*_1_, *Exp*_2_)] was around 3.08%. This result shows that the measurements of properties in the characterization of a manually segmented neuron vary from one neuroanatomist to another and a little bit more from a neuroanatomist to the automatic procedure. This could be due to the neuroanatomist making similar mistakes. Since the proposed method is deterministic and mathematically founded, its application is useful for achieving reproducible and objective results.

### 2.5. Intra-neuroanatomist variability

The cutoffs on neurons are subject to variation due to human inaccuracy and the limitations of the hardware and software used for 3D reconstructions of the cells. For example, the segmentation of three-dimensional meshes on a computer screen changes the morphology of the resulting soma depending on the perspective of the neuron when it is cut. Hence, a neuroanatomist segmenting the same neuron never obtains the same soma. This intra-neuroanatomist variability can be avoided by the proposed procedure, which yields deterministic results.

**Figure 10:**
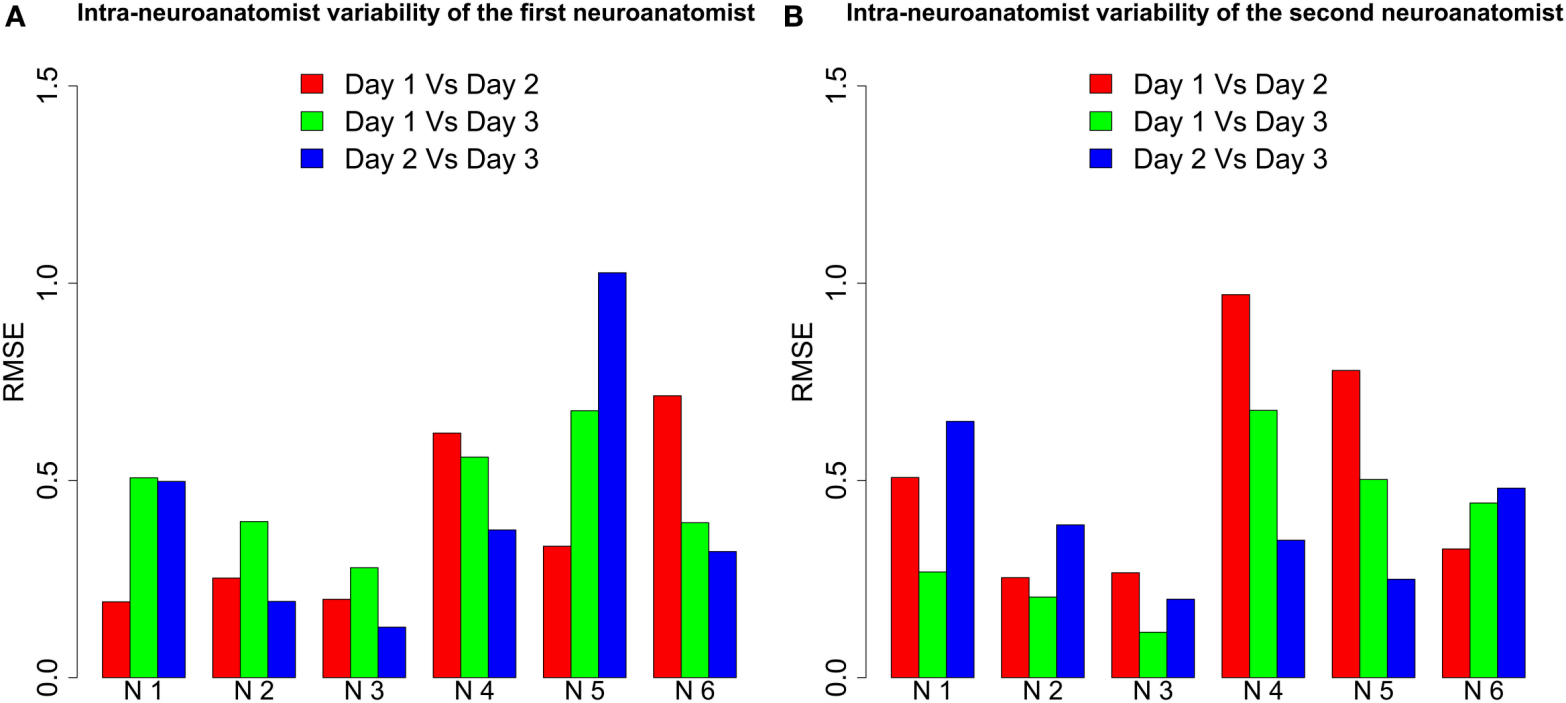
**RMSE between the somata extracted from six neurons by both neuroanatomists on three different days**. We denote each neuron by N followed by a number. **(A)** For the first neuroanatomist, intra-neuroanatomist variability was high for Neurons 4, 5, and 6, whereas Neurons 2 and 3 were quite accurately segmented. **(B)** For the second neuroanatomist Neurons 4 and 5 stand out from the others because of their higher variation. Again Neurons 2 and 3 were the most accurately segmented.

To test this, the two neuroanatomists segmented six repaired neurons three times, each on a different day. The intra-neuroanatomist variability was estimated from these somata. The results are shown in Figure 10. As the bar plot shows, the same neuroanatomist never gets the same result for the same neuron. Additionally, neuroanatomists found some neurons harder to segment. See, for example, Neuron 5 for the first neuroanatomist or Neuron 4 for the second neuroanatomist. However, the intra-neuroanatomist variability is close to the inter-neuroanatomist variability observed in Figure 8. In fact, the mean inter-neuroanatomist RMSE was 0.458, whereas the mean intra-neuroanatomist variability was 0.4254 for the first neuroanatomist and 0.4236 for the second neuroanatomist. Therefore, applying the proposed procedure removed the main factor that induced the intra-neuroanatomist variability originating from the same neuron.

**Figure 11:**
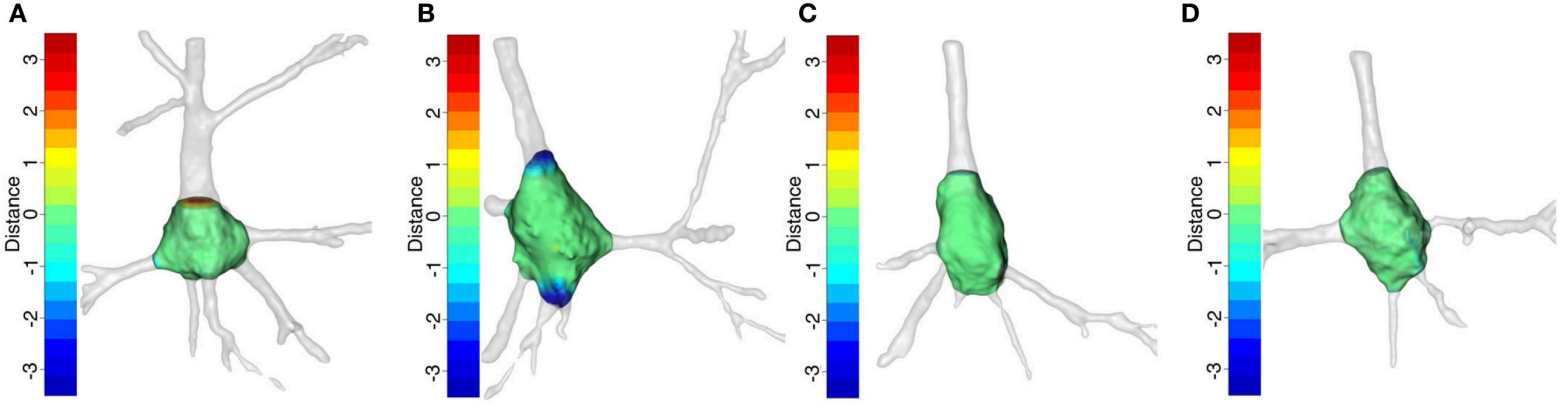
**Somata with their primary dendrites after manual segmentation**. The somata surface is green. The cutoffs are denoted by a color on a scale between red and blue in such a way that the longest distances are denoted by the end colors and the shortest distances by an equal combination of both. The opacity of the primary dendrites was decreased in order to show up the colors of the cutoffs. **(A,B)** illustrate the somata with the greatest differences according to Figure 10, i.e., **(A)** is Neuron 5 segmented by the first neuroanatomist and **(B)** is Neuron 4 segmented by the second neuroanatomist. **(C,D)** show the somata with the smallest differences, i.e., **(C)** is Neuron 2 segmented by the first neuroanatomist **(D)** is Neuron 3 segmented by the second neuroanatomist.

We studied the soma locations at which some neurons were harder to segment than others using the distances between meshes. The R package Morpho (Schlager, 2014) provides a functionality to color a mesh according to its distance to the compared mesh (Figure 11). As a result, easily identifiable cutoffs were shaded green, like the surface of the soma. However, troublesome cutoffs were shaded red when the dendrite was longer than that of the other mesh and blue otherwise. Thus, by exposing the morphology around the soma and combining it with the colors of the cutoffs, the hot spots were highlighted and the causes of differences between cutoffs were analyzed.

Figures 11A,B are the best examples of the differences between the neuroanatomist segmentations. They show that intra-neuroanatomist discrepancies occur in the thickest primary dendrites, especially the apical dendrite. This denotes the intrinsic complexity of segmenting the apical dendrite properly. By contrast, the neurons shown in Figures 11C,D have thinner primary dendrites and are easier to segment, which makes it simpler to get accurate cutoffs.

## 3. Discussion

This paper provides a mathematical definition of the neuronal soma and an automatic segmentation method to delimit the neuronal soma of pyramidal cells, the most abundant neurons in the cerebral cortex (DeFelipe and Fariñas, 1992). Since there are no benchmarks with which to compare the proposed procedure, we validated the goodness of this automatic segmentation method against the manual segmentation performed by neuroanatomists in order to set up a framework for comparison. The results have demonstrated the importance of the repair process. Significant differences were found between the morphology of the cell bodies with and without a reconstructed surface. However, after repairing the surface of the somata, there were no significant differences between automatically and manually segmented somata, i.e., the proposed procedure segments the neurons similarly to how a neuroanatomist would. It also provides univocal, justifiable, and objective cutoffs. The cutoffs on neurons are subject to variation due to human inaccuracy and the limitations of the software used for 3D reconstructions of the cells. Furthermore, manual tracing of individual cells is a time-consuming task. It is, therefore, important to develop automated methods for the morphological analysis of large numbers of neurons to enable high-throughput research. We think that the mathematical definition of the soma of pyramidal cells is an important step not only toward establishing and maintaining effective communication and data sharing between different laboratories, but also for better characterizing these cells. For example, it is well known that these cells are heterogeneous with regard to soma size and shape and different subpopulations of pyramidal cells have different size (e.g., Hendry and Jones, 1983). However, there are no accurate morphometric data, and the data variations between different laboratories may simply reflect the discrepancy regarding the delimitation of the cell body. We submit that an undertaking by different laboratories to use the same methodology to define the soma would have a great impact. The reason is that this information is relevant not only for better characterizing the morphology of these cells in different cortical areas and species but also for annotating and exchanging relevant information for modeling the activity of these cells. For example, the method that we propose will help to generate detailed functional models which may require knowledge of the number and density of axo-somatic synapses, or when quantitative data about relevant molecules playing a key role in the physiology of these cells are critical. For example, to provide the density values along the whole neuronal somatic membrane surface of identified pyramidal cells using specific markers for different voltage-gated ion channels and receptors. Furthermore, microanatomical studies have shown that there are considerable variations in the structure of pyramidal cells across different cortical layers, areas and species, including variations in spine density and spatial distribution, as well as the branching pattern of the dentritic arbors (e.g., Jacobs et al., 2001; Elston, 2002). Previous studies have reported variations in the size of pyramidal neurons, but these studies are based on arbitrary soma measurements, impeding comparisons between different laboratories or the performance of other correlational studies, such as the possible relationship between the size of the soma and number of branches, nodes, etc., of the dendritic tree. Thus, this study is an excellent means for further characterizing pyramidal neurons in order to objectively compare the morphometry of the somata of these neurons in different cortical areas and species and try to find possible rules governing the geometric design of pyramidal cells. In particular, this method could be especially useful for the high-throughput characterization of the somata of particular pyramidal cells in transgenic mice expressing multiple spectral variants of green fluorescent protein that label different subpopulations of pyramidal cells (Feng et al., 2000). Furthermore, the proposed method could be applied to any cell (e.g., interneurons and glial cells) labeled with fluorescent dyes or expressing different fluorescent proteins. Future applications of the method would also include the segmentation and analysis of images from conventional 3D light microscopy. Finally, the software of the proposed method and a user's guide are available at CIG web^1^, in the software section, for use by those potential users interested in applying the present algorithm. It should be noted that the original generated triangular meshes necessary for the development of the present method, were created to obtain a single coarse solid surface of a particular threshold, which included both the soma and proximal dendrites of labeled neurons. Thus, they are available only for reproducibility purposes of the present algorithm.

### Conflict of interest statement

The authors declare that the research was conducted in the absence of any commercial or financial relationships that could be construed as a potential conflict of interest.

## Footnotes

^1^http://cig.fi.upm.es/
